# Levodopa Modulates Functional Connectivity in the Upper Beta Band Between Subthalamic Nucleus and Muscle Activity in Tonic and Phasic Motor Activity Patterns in Parkinson’s Disease

**DOI:** 10.3389/fnhum.2019.00223

**Published:** 2019-07-02

**Authors:** Uri E. Ramirez Pasos, Frank Steigerwald, Martin M. Reich, Cordula Matthies, Jens Volkmann, René Reese

**Affiliations:** ^1^Department of Neurology, University Hospital Würzburg, Würzburg, Germany; ^2^Department of Neurosurgery, University Hospital Würzburg, Würzburg, Germany; ^3^Department of Neurology, University of Rostock, Rostock, Germany

**Keywords:** Parkinson’s disease, subthalamic nucleus, deep brain stimulation, local field potentials, dopamine, movement

## Abstract

**Introduction**: Striatal dopamine depletion disrupts basal ganglia function and causes Parkinson’s disease (PD). The pathophysiology of the dopamine-dependent relationship between basal ganglia signaling and motor control, however, is not fully understood. We obtained simultaneous recordings of local field potentials (LFPs) from the subthalamic nucleus (STN) and electromyograms (EMGs) in patients with PD to investigate the impact of dopaminergic state and movement on long-range beta functional connectivity between basal ganglia and lower motor neurons.

**Methods**: Eight PD patients were investigated 3 months after implantation of a deep brain stimulation (DBS)-system capable of recording LFPs *via* chronically-implanted leads (Medtronic, ACTIVA PC+S^®^). We analyzed STN spectral power and its coherence with EMG in the context of two different movement paradigms (tonic wrist extension vs. alternating wrist extension and flexion) and the effect of levodopa (L-Dopa) intake using an unbiased data-driven approach to determine regions of interest (ROI).

**Results**: Two ROIs capturing prominent coherence within a grand average coherogram were identified. A trend of a dopamine effect was observed for the first ROI (50–150 ms after movement start) with higher STN-EMG coherence in medicated patients. Concerning the second ROI (300–500 ms after movement start), an interaction effect of L-Dopa medication and movement task was observed with higher coherence in the isometric contraction task compared to alternating movements in the medication ON state, a pattern which was reversed in L-Dopa OFF.

**Discussion**: L-Dopa medication may normalize functional connectivity between remote structures of the motor system with increased upper beta coherence reflecting a physiological restriction of the amount of information conveyed between remote structures. This may be necessary to maintain simple movements like isometric contraction. Our study adds dynamic properties to the complex interplay between STN spectral beta power and the nucleus’ functional connectivity to remote structures of the motor system as a function of movement and dopaminergic state. This may help to identify markers of neuronal activity relevant for more individualized programming of DBS therapy.

## Introduction

*In vivo* recordings of basal ganglia neuronal activity in Parkinson’s disease (PD) patients have been facilitated by the chronic implantation of stimulation electrodes for therapeutic deep brain stimulation (DBS; Benabid et al., 1994). Local field potential (LFP) recordings from the subthalamic nucleus (STN), the most common DBS target for PD (Deuschl et al., 2006; Weaver et al., 2009; Follett et al., 2010; Schuepbach et al., 2013), have revealed abnormally synchronized oscillatory activity in the beta band (about 13–30 Hz) to be an electrophysiological hallmark of the Parkinsonian state (Brown et al., 2001; Brown, 2003; Kühn et al., 2006, 2009). STN beta activity is most prominent in the resting state of PD patients (López-Azcárate et al., 2010), is attenuated by voluntary movement (Cassidy et al., 2002; Levy et al., 2002; Priori et al., 2002; Kühn et al., 2004; Alegre et al., 2005, 2013; Doyle et al., 2005; Foffani et al., 2005; Hebb et al., 2012; Joundi et al., 2012, 2013; Litvak et al., 2012), and increases again when movement voluntarily ends (Kühn et al., 2004; Alegre et al., 2013). It has been suggested that the encoding of steady motor states might be a physiological role of beta rhythms, which become pathologically exaggerated as a result of striatal dopamine depletion, a pathophysiological hallmark of PD (Engel and Fries, 2010; Brittain and Brown, 2014). This mechanism is supported by several studies demonstrating that oral levodopa (L-Dopa) intake in PD patients desynchronizes STN beta band activity (Brown et al., 2001; Levy et al., 2002; Priori et al., 2004; Alegre et al., 2005; Kühn et al., 2006; Hirschmann et al., 2013). Beta desynchronization is moreover correlated with improved bradykinesia scores (Kühn et al., 2006, 2009; Weinberger et al., 2006; Androulidakis et al., 2008; Ray et al., 2008). Because of the correlative nature of these experiments, it remains an open question whether STN beta rhythms cause bradykinesia (Jenkinson and Brown, 2011), despite ample evidence that beta exaggerations represent a valid electrophysiological marker of the Parkinsonian motor OFF state. Studies which evaluated the effects of dopamine and movement on functional beta oscillatory coupling of STN, motor cortex, and muscle activity have delivered conflicting results (Marsden et al., 2001; Salenius et al., 2002; Litvak et al., 2012; Hirschmann et al., 2013), possibly due to differences in recording and analyses techniques as well as movement paradigms. Frequency-specific coding of STN-muscle coherence was found in the beta band during isometric contractions and in the 31–45 Hz band during phasic motor activity in PD patients after L-Dopa intake (Marsden et al., 2001). In contrast, Hirschmann et al. (2013) found reduced STN-muscle beta band coherence during alternating forearm movements compared to isometric contractions, which was independent of L-Dopa medication. One study found STN-motor cortex beta coherence in a task of tonic muscle activity to be greater compared to that of alternating movements in the L-Dopa ON state of PD patients (Marsden et al., 2001), whereas other studies found this coherence suppressed (Hirschmann et al., 2013) or restored by L-Dopa during movement (Salenius et al., 2002). With a latency of 2 s from movement onset, STN-motor cortex beta band coherence increased compared to baseline, which was independent of dopaminergic medication state (Litvak et al., 2012). In contrast, STN spectral power and STN-motor cortex coherence in the beta band decreased compared to baseline prior to and during movement in unmedicated PD patients (Talakoub et al., 2016). Only these two studies have explicitly looked at dynamic changes of STN oscillatory activity and STN-cortex coherence to be dependent on movement activity. This is of considerable significance given that increased beta oscillations in motor cortex and basal ganglia have been associated with immutability and steady state in motor activity while being modulated during movement change (Brittain and Brown, 2014) Additionally, the analysis of dynamic changes in oscillatory beta band activity and coherence between relay structures of the motor circuit may be relevant in the process of defining physiological read-out parameters for on demand stimulation systems as closed loop techniques.

In the above-mentioned studies, recordings in the STN were performed through externalized leads in the immediate post-operative period, when a surgical stun effect may mitigate the Parkinsonian OFF-state. Here, we investigated patients implanted with the ACTIVA PC+S^®^ (Medtronic Inc., Minneapolis, MN, USA), a DBS device that is capable of recording LFP activity from chronically implanted STN leads, and studied the effect of motor task and dopaminergic state on the modulation of beta band activity using an event-related approach in PD patients. We were interested to test whether the STN differentially encodes movements that involve changes (here the voluntary alternating movement) and movements that do not involve changes (here the isometric contraction), and if so, where in time-frequency this differential processing is localized.

## Patients and Methods

### Patient Characteristics and DBS Device

The study protocol was approved by the ethical review board of the University Hospital Würzburg and patients provided written informed consent to participate prior to implantation. We investigated eight PD patients with the following characteristics: mean age 58 years (range: 50–66), one female, one left-handed, mean disease duration 13 years (range: 10–19), mean pre-operative/post-operative L-Dopa-equivalent-dose: 1295 mg/505 mg (range: 650–2725/180–800), mean UPDRS III pre-operative L-Dopa OFF/pre-operative L-Dopa ON/post-operative L-Dopa OFF stimulation ON: 49 (range: 40–69)/15 (range: 4–24)/14 (range: 9–19). Patients were selected for STN-DBS according to established clinical criteria and implanted with a CE-approved DBS device (ACTIVA PC+S^®^ Medtronic Inc., Minneapolis, MN, USA), which has the capability of recording LFP *via* standard, quadrupolar DBS electrodes (3389, Medtronic Inc., Minneapolis, MN, USA). Data can be stored within the device and downloaded later for off-line analysis. The implantation of the DBS system was performed according to standard clinical routine as described previously (Steigerwald et al., 2008; Reese et al., 2012).

### Experimental Procedure

The study was performed 3.5 ± 0.2 months after DBS implantation when stimulation parameters and dopaminergic medication had been optimized and a stable improvement of PD motor symptoms had been achieved. Long-acting dopamine agonists were suspended for about 3 days and L-Dopa was withdrawn overnight at least 12 h before recording the dopamine depleted state (L-Dopa OFF), while the L-Dopa-medicated state (L-Dopa ON) was recorded 1 h after intake of 200 mg of soluble L-Dopa/benserazide (Madopar LT^®^, Hoffmann-La Roche AG, Switzerland). Patients were required to fast prior to medication. Recordings were performed subsequently on the same day in L-Dopa OFF and ON condition and STN-DBS remained switched off during all experimental procedures.

Only the brain hemisphere with the most prominent STN beta band activity in the L-Dopa OFF resting state (right *n* = 4, left *n* = 4) and the contralateral bodyside was evaluated.

Patients were comfortably seated and asked to position the evaluated forearm onto a table in front of them, while the contralateral arm rested in their lap.

STN-LFP were recorded (sampling rate: 794 Hz, gain: 1,000 ×) from two adjacent electrode contacts with most prominent beta activity and which were clinically effective. These contacts were located at the dorsolateral STN as determined by an image fusion of the pre-operative magnetic resonance imaging (MRI)- and post-operative CT-scan (Optivise^®^ Software, Medtronic Inc., Minneapolis, MN, USA). Electromyogram (EMG; sampling rate: 1 kHz, 7–500 Hz anti-aliasing band-pass filter) was recorded *via* two adhesive surface electrodes (1 cm diameter, Ag/AgCl, Zebris Medical GmbH, Germany) positioned on the wrist extensor muscles. For subsequent alignment of EMG and LFP recordings, a brief high-frequency electrical train pulse was applied *via* a standard TENS device (transcutaneous electrical nerve stimulation) at the start and end of each recording, creating a stimulus artifact in the LFP and EMG recordings.

Two simple motor tasks were performed in random order, each three times for at least 30 s: submaximal tonic = isometric wrist extension (ISO) or self-paced, alternating wrist flexion and extension (ALT).

### Signal Analysis

#### Preprocessing

STN-LFP and EMG recordings were inspected offline for artifacts using Spike2 software (CED, Cambridge, UK). EMG signals were downsampled to 794 Hz and aligned with STN-LFP recordings based on the sharp onset of the TENS artifact. Data were then further processed in MATLAB (Release 2016b, The MathWorks, Inc., Natick, MA, USA). Movement onset and stop was detected by a threshold method on the z-transformed envelope of the band-pass filtered EMG (Butterworth filter of 4th order with cut-off frequencies 110 Hz and 140 Hz) and visually confirmed. STN-LFP and EMG recordings were band-pass filtered between 1 Hz and 90 Hz using an IIR Butterworth filter of 4th order using a forward and a reverse pass. Both recording types were then baseline-corrected *via* simple subtraction of mean voltage between −4 s and −2 s relative to movement onset. Epochs of ±5 s duration were extracted relative to movement onset.

#### Long-Range Coupling and STN Spectral Power

STN-muscular functional connectivity was evaluated by means of wavelet coherence, which is a measure of amplitude and of the consistency of phase relationships between STN-LFP vs. EMG across time (Grinsted et al., 2014). EMG was not rectified since rectification leads to inconsistencies in the extraction of oscillatory content in muscle activity in the presence of varying levels of contraction (Farina et al., 2013; McClelland et al., 2014). EMGs were not baseline-corrected in the frequency domain. The fact that statistical properties of the EMG are expected to vary across time during changes in muscular activity does not pose a problem when using wavelet coherence (as opposed to Fourier coherence) since wavelet methodology does not presuppose the underlying signals to be stationary (Zhan et al., 2006). Coherence values were obtained using Morlet wavelets by means of MATLAB’s wavelet coherence function (with ω_0_ = 6) as a trade-off between time and frequency localization (van Vugt et al., 2007). Wavelet coherence values were trial-averaged for each subject and condition (ALT OFF, ALT ON, ISO OFF, ISO ON).

STN-LFP power spectral density values were computed *via* Fieldtrip’s ft_frequency analysis function using Hanning windows as tapers before performing a Fourier transformation (Oostenveld et al., 2011). In order to perform both regions of interest (ROI)-based analysis of variance and permutation tests comparing the pre-movement baseline to movement, power values were computed for frequencies 4–50 Hz with a resolution of 1 Hz, with time window length of 500 ms and a step size of 50 ms. Recordings were analyzed from −5 s to 5 s relative to movement onset.

### Statistics

An unbiased, data-driven ROI was identified as the largest region of coherence within the confines of 0–1 s and 1–50 Hz on the mean coherogram across subjects of the mean coherogram across the four conditions. ROIs were selected by drawing a quadrilateral over the prominent regions of coherence visible on the grand average map after masking away coefficients under 0.3. Examining the average across conditions precludes double dipping (Kriegeskorte et al., 2009). Mean coherence values within the identified ROI were subsequently extracted for each patient and condition. The effects of dopamine and movement type on STN-EMG coherence were analyzed using a two-way analysis of variance (ANOVA) with repeated measures for both fixed effects, namely DOPAMINE (with levels OFF and ON) and MOVEMENT (with levels ALT and ISO) that included SUBJECTS as a random effect. In order to examine whether dopamine and movement type effect on STN-EMG coherence by parallel, frequency-specific modulation of STN spectral power and EMG activity, the same ROI was employed on trial-averaged power spectral density data and analyzed using the same factorial design as above. For this, trial-averages were baseline-corrected by computing the decibel changes from baseline (−4 to −2 s relative to movement onset). Statistical significance was set at *p* = 0.05/(number of ROIs) to correct for multiple comparisons.

In addition, movement-induced changes in STN spectral power were compared to baseline levels *via* nonparametric permutation testing (Theiler et al., 1992; Maris and Oostenveld, 2007; MATLAB code adapted from Cohen, 2014). The goal was to create a null hypothesis distribution to compare our observed data to. Here, the null hypothesis states that movement initiation induces no difference in STN spectral power with respect to baseline, and therefore any temporal reordering of the data should possess statistical properties similar to those of the observed dataset. Prior to permutation, power maps were smoothed with a 2-D Gaussian kernel (σ = 2). The baseline vs. movement comparison was conducted for each condition separately, as well as after averaging across conditions for each subject. First, an average map across subjects (the “observed map”) was computed and was baseline-corrected by obtaining the decibel change from its baseline. Each permutation was obtained by randomly selecting a timepoint *t* within the epoch, then dividing each (uncorrected) subject map at *t* into two submaps and placing the earlier submap after the later submap, thus essentially randomly shifting the subject maps along the time axis. Time-shifted subject maps were then averaged and subsequently baseline-corrected by computing the decibel change from the baseline values of the observed map. This process was repeated 1,000 times to have a null hypothesis distribution of power values for each time-frequency bin. To determine statistical significance, a z-map was computed by bin-wise subtracting the mean of the distribution from the observed map and dividing by the standard deviation of the distribution. The z-map was thresholded such that only *z*-scores corresponding to a *p*-value of 0.01 or less survived. The *p*-value threshold was chosen to account for multiple comparisons (here 4). The ensuing clusters of significant points were corrected by eliminating clusters with pixel sizes lower than a cluster size threshold defined by the permuted data. To derive such threshold, a distribution of maximum cluster sizes was obtained as follows. Each permutation was standardized by subtracting the mean permutation map and dividing by its standard deviation, yielding z-maps which were thresholded as above, after which the maximum cluster size was stored. The cluster size threshold was defined as equal or greater than the 99th percentile of the distribution of largest clusters.

## Results

**Figure 1:**
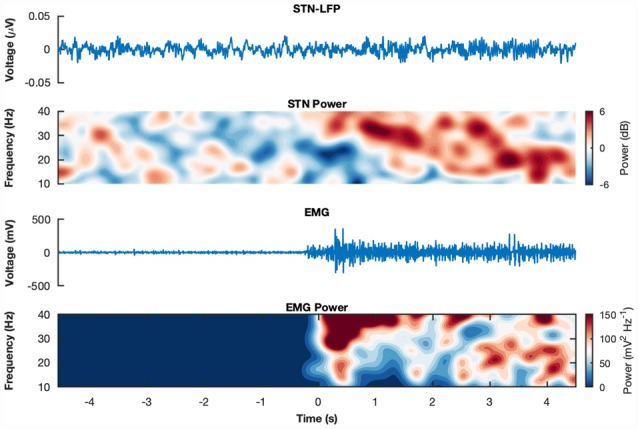
Raw recordings and spectrograms. Example raw subthalamic nucleus (STN)-Local Field Potentials (LFPs) and electromyogram (EMG) epochs from subject wue11 during an isometric contraction in medication state ON. The corresponding spectrograms display the trial-average power spectral densities for the same subject and condition for the STN-LFP and the EMG, respectively. The spectrograms show the decibel change from baseline (−4 s to −2 s relative to movement onset).

3.5 months after implantation all patients presented an excellent response to STN-DBS reaching at least 80% of the pre-operative UPDRS-III L-Dopa-ON score. One patient (ID 4) was excluded from the analysis since she/he could not complete the whole experiment due to severe OFF motor symptoms. Two trials from subject-ID 3 were rejected after visual inspection due to electrical artifacts. Total number of ISO trials were *n* = 21 for L-Dopa-OFF and *n* = 20 for L-Dopa-ON. For ALT trials *n* = 20 for L-Dopa-OFF and *n* = 21 for L-Dopa-ON. Exemplary raw STN-LFP and EMG epochs and corresponding spectrograms can be seen in Figure 1.

### STN-Muscular Coherence

**Figure 2:**
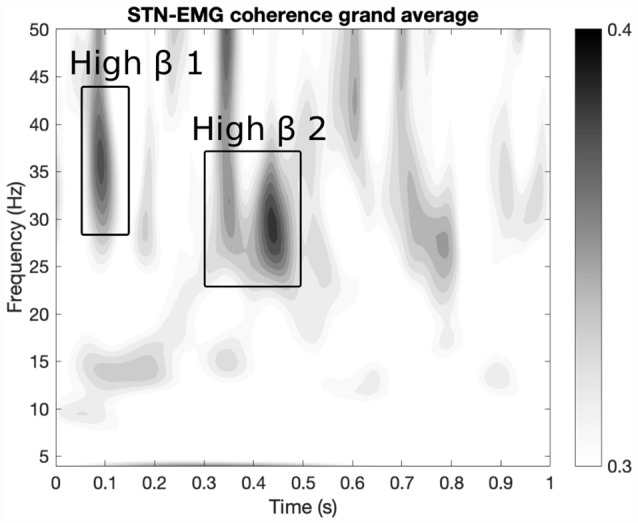
Regions of interest (ROIs) extraction. Two ROIs were extracted from the grand average map of STN-EMG coherence. The map displays the average coherence across all conditions within the ranges 0–1 s and 4–50 Hz. Two sizeable ROIs were identified: “high beta 1” within 28–44 Hz and 50–150 ms and “high beta 2” enclosed by the ranges 23–37 Hz and 300–500 ms.

**Figure 3:**
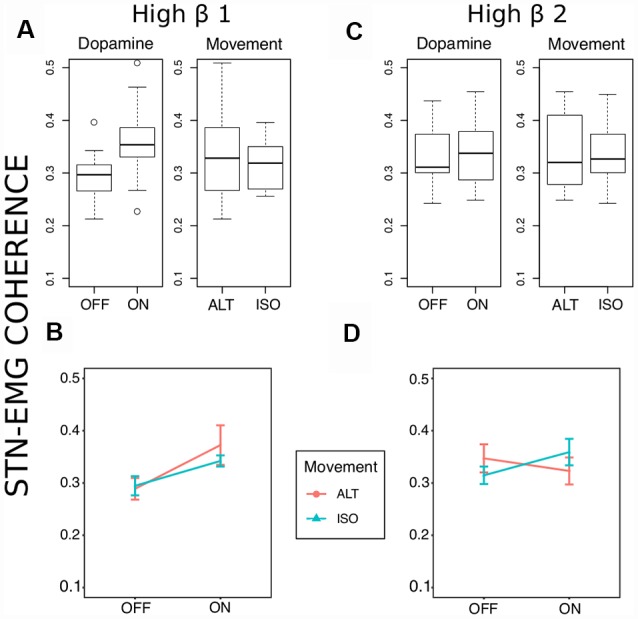
Coherence statistics. **(A,B)** Stronger high beta coherence soon after movement onset (50–150 ms) was observed in the medication ON state, though this was only marginally significant (*F*_(1,6)_ = 5.41, uncorrected *p* = 0.059, $\eta_{\text{p}}^{2}$ = 0.47). **(C,D)** A significant dopamine × movement type effect (*F*_(1,6)_ = 9.37, uncorrected *p* = 0.02, $\eta_{\text{p}}^{2}$ = 0.61) on high beta coherence was observed later between 300 and 500 ms after movement onset.

Two prominent regions of STN-EMG coherence were identified, namely “high beta 1” in the frequency range 28–44 Hz soon after movement onset (50–150 ms) and “high beta 2” enclosed by the ranges 23–37 Hz and 300–500 ms (Figure 2). Shapiro-Wilk tests on each model’s residuals yielded *W* = 0.96, *p* = 0.37 and *W* = 0.95, *p* = 0.19, respectively. The results of the repeated-measures ANOVAs can be seen in Table 1. We conducted a two-way repeated-measures ANOVA to compare the effect of dopamine and movement type on STN-EMG coherence. Coherence in high beta 1 showed a DOPAMINE trend (*F*_(1,6)_ = 5.4, *p* = 0.059) and no significant MOVEMENT or interaction effects. In contrast, high beta 2 yielded a significant DOPAMINE × MOVEMENT interaction (*F*_(1,6)_ = 9.37, *p* = 0.022; Figure 3), wherein the OFF state coherence is higher in ALT (0.35 ± 0.03) than in ISO (0.31 ± 0.02), whereas in the ON state functionally connectivity was stronger in ISO (0.36 ± 0.03) than in ALT (0.32 ± 0.03). STN-EMG wavelet coherence was not significantly modulated by dopamine or movement type.

**Table 1:** Summary of statistics of subthalamic nucleus (STN) and electromyogram (EMG) coherence and STN power.

	STN-EMG Coherence		STN Power (dB)	
	High β1	High β2	High β1	High β2
*Dopamine*				
OFF	0.29 ± 0.01	0.33 ± 0.02	−1.4 ± 0.36	−2.02 ± 0.53
ON	0.36 ± 0.02	0.34 ± 0.02	−0.09 ± 0.43	−0.69 ± 0.48
	*F*_(1,6)_ = 5.41	*F*_(1,6)_ = 0.12	*F*_(1,6)_ = 3.65	*F*_(1,6)_ = 2.27
	*p* = 0.059	*p* = 0.75	*p* = 0.10	*p* = 0.18
	$\eta_{\text{p}}^{2}$ = 0.47	$\eta_{\text{p}}^{2}$ = 0.02	$\eta_{\text{p}}^{2}$ = 0.38	$\eta_{\text{p}}^{2}$ = 0.27
*Movement type*
ALT	0.33 ± 0.02	0.34 ± 0.02	−0.29 ± 0.42	−1.13 ± 0.58
ISO	0.32 ± 0.01	0.34 ± 0.02	−1.2 ± 0.42	−1.58 ± 0.48
	*F*_(1,6)_ = 0.26	*F*_(1,6)_ = 0.005	*F*_(1,6)_ = 6.73	*F*_(1,6)_ = 2.61
	*p* = 0.63	*p* = 0.95	*p* = 0.041	*p* = 0.16
	$\eta_{\text{p}}^{2}$ = 0.04	$\eta_{\text{p}}^{2}$ < 0.01	$\eta_{\text{p}}^{2}$ = 0.53	$\eta_{\text{p}}^{2}$ = 0.30
*Dopamine × Movement type*				
OFF ALT	0.29 ± 0.02	0.35 ± 0.03	−1.2 ± 0.43	−2.1 ± 0.88
ON ALT	0.37 ± 0.04	0.32 ± 0.03	0.59 ± 0.56	−1.15 ± 0.61
OFF ISO	0.30 ± 0.02	0.31 ± 0.02	−1.6 ± 0.61	−1.93 ± 0.67
ON ISO	0.34 ± 0.01	0.36 ± 0.03	−0.78 ± 0.58	−1.23 ± −73
	*F*_(1,6)_ = 1.08	*F*_(1,6)_ = 9.37	*F*_(1,6)_ = 0.51	*F*_(1,6)_ = 1.32
	*p* = 0.34	*p* = 0.02*	*p* = 0.5	*p* = 0.3
	$\eta_{\text{p}}^{2}$ = 0.15	$\eta_{\text{p}}^{2}$ = 0.61	$\eta_{\text{p}}^{2}$ = 0.08	$\eta_{\text{p}}^{2}$ = 0.18

*Statistics. ROI values for STN-EMG coherence and baseline-corrected STN power shown as mean ± SEM, and statistics for each effect of the repeated measures ANOVAs. An asterisk indicates statistical significance, which was set at p = 0.025 (p-values shown uncorrected)*.

### STN Spectral Power

**Figure 4:**
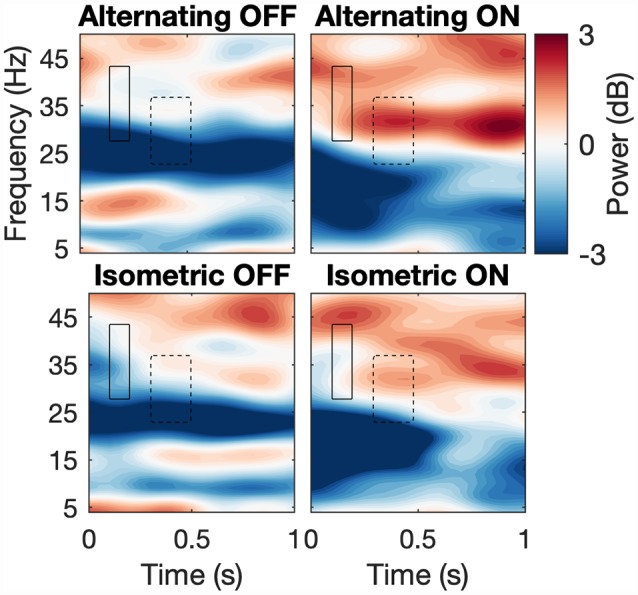
Spectrograms across conditions. Average spectrograms across subjects depicting first second after movement onset for each condition with ROIs. Hard line and dashed line correspond to high beta 1 and 2, correspondingly.

**Figure 5:**
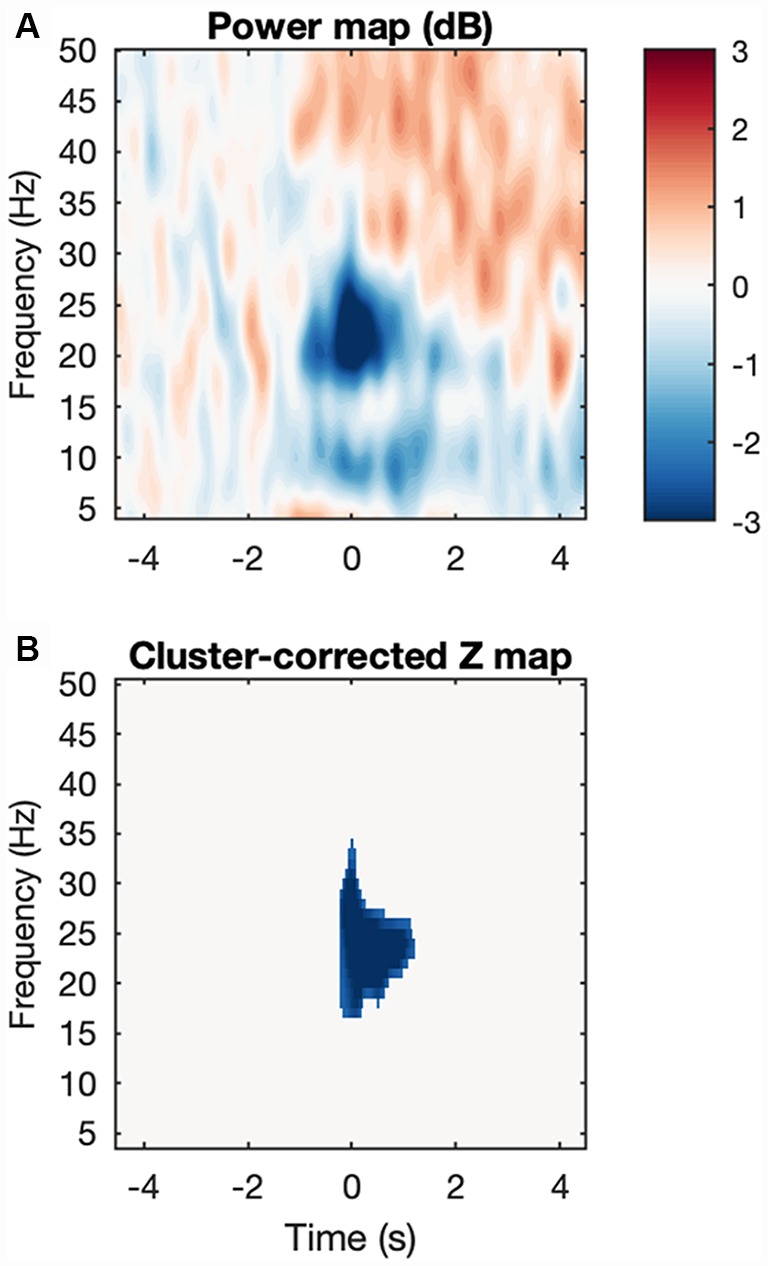
Movement-induced high beta desynchronization. **(A)** Baseline-corrected grand average map, where each subject map was the average across all four conditions. **(B)** After cluster correction, a final cluster survives showing event-related desynchronization and located at about −50 to 1,000 ms and 18–27 Hz.

We analyzed the same ROIs with the power data using the same design as above (Figure 4). For STN-LFPs, Shapiro-Wilk’s tests on residuals yielded *W* = 0.97, *p* = 0.55 and *W* = 0.95 *p*-value = 0.23, respectively. However, no effects were significant for both ROIs. For EMG synchronization, normality of the residuals was violated for both ROIs (Shapiro-Wilk tests, where high beta 1: *W* = 0.78, *p* = 5.645e-05; high beta 2: *W* = 0.87, *p* = 0.0019). As expected, the permutation test for subject-average maps showed a significant cluster of high beta (about 18–28 Hz) desynchronization starting just before movement onset (about −50 ms to 1,000 ms; Figure 5). However, among the different conditions, this pattern was only significant at the onset of the alternating movement in the ON state (data not shown). Interestingly, a pattern of high beta (about 23–33 Hz) resynchronization is observable 1,000–3,500 ms after movement onset in the ON state for both movement types but not in the OFF state. A two-way ANOVA designed as above threw no significant effects (data not shown).

## Discussion

We assessed the STN-LFP spectral power and STN-LFP-EMG coherence of two different movement patterns after movement onset, namely ISO and alternating wrist flexion and extension, as well as the effect of L-Dopa medication in PD patients. Our finding of movement-related beta desynchronization within the STN is not surprising and is in agreement with numerous studies describing beta oscillations as corresponding to antikinetic activity (Cassidy et al., 2002; Levy et al., 2002; Priori et al., 2002; Kühn et al., 2004; Alegre et al., 2005, 2013; Doyle et al., 2005; Foffani et al., 2005; Hebb et al., 2012; Joundi et al., 2012, 2013; Litvak et al., 2012). Using an unbiased, data-driven approach, we identified two ROIs in the upper beta band within the first second after movement onset (Figure 2). At 300–500 ms, we found a significant DOPAMINE × MOVEMENT interaction for coherent activity, with greater upper beta band coherence in the isometric contraction task compared to alternating wrist movements in the L-Dopa medicated state. This pattern might go awry in the unmedicated state, where coherence is higher for alternating wrist movements. As the peak amplitude of EMG activity occurred at 555 ms after movement onset, i.e., about 50 ms after our defined ROI ends, the prominent STN-EMG coherence cannot be solely traced back to the peak EMG amplitude. The identified ROIs turned out to be after movement onset, where the realized movement is identical between ISO and ALT. In the STN, activity at this short period may still be differently encoded if the STN also encodes whether the movement itself involves change or not. That is, ALT and ISO can be considered (1) each a unit of ongoing contractions which are different from rest (hence beta desynchronization at movement onset), or (2) they can, in addition, be encoded as a series of subunits if the movement itself involved different phases (as is the case in ALT). If (2) is true, then the STN activity may represent ALT and ISO differently *via* differential spectral power profiles. However, because the identified ROIs do take place after movement onset, one could argue that the prominent regions of STN-EMG coherence could reflect basal ganglia movement control (Graybiel et al., 1994; Yin, 2014).

### Methodological Considerations and Possible Confounders

The recordings were obtained several weeks after implantation of the electrodes and therefore rapid impedance changes are unlikely and should not have affected data quality (Rosa et al., 2010). The micro-lesioning effect due to insertion of the electrodes into the STN, which often attenuates Parkinsonian motor symptoms and naturally interferes with stimulation and medication effects, had abated at the time of the experiment as evidenced by patients reaching their pre-operative symptom severity after withdrawal of medication and DBS. We recorded LFPs in a bipolar montage from adjacent contacts with most prominent beta activity during a resting state in L-Dopa-OFF, which may indicate the motor part of the STN (Yoshida et al., 2010; Accolla et al., 2016; Horn et al., 2017). These contacts were located at the dorsolateral aspect of the STN according to an image fusion of the pre-operative MRI and the post-operative CT-scan (Optivise^®^ software, Medtronic Inc., Minneapolis, MN, USA) and were of good clinical efficacy of STN-DBS. Nevertheless, this is a pragmatic and mostly electrophysiologically-driven delineation of the STN motor region, which may not correspond to the “true” physiological motor area in individual patients. Likewise, the low number of patients and trials may have led to reduced sensitivity, however, we draw this sample given by the restricted availability of the PC + S stimulation systems.

### Subthalamic Nucleus-Muscle Coherence (STN-Muscle Coupling)

Within the two identified ROIs a trend of the dopamine effect was observed for the first ROI (50–150 ms) with higher coherence in medicated patients (Figures 2, 3). Concerning the second ROI (300–500 ms), an interaction effect of L-Dopa medication and movement task was significant with higher coherence in the isometric contraction task compared to alternating movements, an effect which was reversed in the dopaminergic OFF state. Owing to the fact that EMG recordings reflect lower motor neuron synchronization only after movement start, we cannot compare our data to the pre-movement period. In a study employing a comparable movement task, the coherence magnitude between the motor cortex and the STN in the upper beta band was markedly attenuated during movement (Talakoub et al., 2016).

### Movement-Related STN Spectral Power

The previously mentioned increase in beta desynchronization in the L-Dopa ON state compared to the OFF state (Doyle et al., 2005; Devos et al., 2006; Androulidakis et al., 2007) did not reach a level of significance in our study, which could be attributed to a low signal-to-noise ratio and sample size. Likewise, no significant influence of the movement task could be detected. Joundi et al. (2013) could show that a finger tapping induced suppression of STN spectral power may be independent of the velocity of these repetitive movements and an inter-movement rebound of beta synchronization may only be suppressed at higher tapping rates. The degree of desynchronization has moreover been shown to depend on the effort of the respective motor task, which may reach a floor effect at higher efforts. The latter strongly correlated with spectral power in the gamma band (55–90 Hz; Tan et al., 2013, 2015). Attenuated cortical post-movement beta synchronization has been shown in bradykinetic-rigid PD compared to healthy controls (Pfurtscheller et al., 1998; Tamás et al., 2003). In our data, high beta (about 23–33 Hz) resynchronization in the STN about 1,000–3,500 ms after movement onset was stronger in the ON than in the OFF medication state, although not significantly so (Figure 4).

### Physiological Considerations

Synchronized oscillatory activity between remote neuronal populations can facilitate information processing over large distances (Varela et al., 2001; Schnitzler and Gross, 2005). There is good evidence that motor system communication over large distances is physiologically realized *via* the segregation of synchronization in distinct frequency bands. In this view, motor cortical synchronization in the beta band was initially regarded as representing “idleness” (Pfurtscheller et al., 1996) but it may instead be conductive to motor constancy (Brittain and Brown, 2014). For instance, studies have shown that movement onset is accompanied by desynchronization of motor cortical beta activity (event-related desynchronization, ERD) with rebound synchronization around movement stop (Pfurtscheller and Lopes da Silva, 1999). In PD patients, the motor cortical pre-movement ERD is delayed and its hemispheric specificity is reduced compared to healthy controls (Defebvre et al., 1993, 1994, 1996; Magnani et al., 1998). Exaggerated beta synchrony is therefore strongly associated with the OFF (dopamine depleted) motor state (Jenkinson and Brown, 2011). Conversely, post-movement beta synchronization (PMBS) is attenuated in PD (Pfurtscheller et al., 1998; Tamás et al., 2003), which may be a marker of certainty in feedforward control of movements. In healthy subjects, PMBS has been shown to be negatively correlated with uncertainty in feedforward estimations (Tan et al., 2016).

We used PD as a model disease to further investigate the role of dopamine in the context of motor activity and we reproduced a movement-dependent ERD of STN beta activity (Cassidy et al., 2002; Levy et al., 2002; Priori et al., 2002; Kühn et al., 2004; Alegre et al., 2005, 2013; Doyle et al., 2005; Foffani et al., 2005; Hebb et al., 2012; Joundi et al., 2012, 2013; Litvak et al., 2012), whose magnitude, however, was not significantly affected by dopamine substitution. Long-range STN-muscle coherence in the upper beta band increased shortly after movement onset and a trend of a dopamine effect could be observed with stronger functional connectivity in the ON state. Thus, beta spectral power *per se* might not simply be a universal marker of bradykinesia. Instead, our data suggest STN beta spectral power to be present at motor rest and reduced with changes in motor activity (ERD) while a subset of neurons, however, functionally connects to the spinal motor neuron pool (measured by EMG activity). Only after substitution of L-Dopa was STN-muscle coherence in the upper beta band stronger in the isometric contraction task compared to alternating movements. Assuming that this state is closer to normal motor function than in PD patients off L-Dopa medication, beta coherence could physiologically limit the amount of information conveyed between remote structures, which may be necessary to maintain simple movements like the isometric contraction. In this view, a physiologically inverse relationship between neuronal synchrony and information flow has been hypothesized (Hanslmayr et al., 2012; Oswal et al., 2013). Our data also suggest that higher striatal dopamine levels may be essential for an appropriate setting of neuronal synchrony in the STN and information flow between remote structures of the motor circuit. Salenius et al. (2002) reported a similar effect of L-Dopa with restorage of cortico-muscular beta coherence in PD patients during the isometric contraction of contralateral forearm muscles.

Muscle activity synchronized in higher frequencies of about 40 Hz (called Piper-rhythm, see Piper, 1912) can be recorded from muscles of normal subjects during muscle activity near maximum contraction force (McAuley et al., 1997). In PD patients, impaired muscle strength in the medication OFF state (Yanagawa et al., 1990; Corcos et al., 1996) is correlated with a disruption of this higher frequency synchronization patterns, both of which return to normal levels in patients after L-Dopa substitution (McAuley et al., 2001). Accordingly, the basal ganglia-lower motor neuron coupling detected in our study may be a physiological phenomenon necessary for normal motor execution. Consequently, impaired coupling on various relay stations of the motor circuit in PD may contribute to Parkinsonian OFF motor symptoms, such as bradykinesia, hypokinesia and reduced muscle strength.

A simple monosynaptic connection between basal ganglia nuclei and spinal motor neurons is unknown (Lanciego et al., 2012), thus anatomical pathways explaining our findings have to be discussed. Motor cortical beta oscillatory activity has been shown to drive beta oscillations in the STN (Williams et al., 2002; Fogelson et al., 2006; Litvak et al., 2011), most likely *via* the so-called “hyperdirect pathway” (HDP; Monakow et al., 1978; Nambu et al., 1996, 1997). Recently, the HDP has also been uncovered and further characterized in humans (Brunenberg et al., 2012; Miocinovic et al., 2018). This pathway may represent the anatomical correlate of clinical efficacy of STN-DBS which may act *via* a retrograde propagation of DBS signals to the motor cortex (Gradinaru et al., 2009; Li et al., 2012; de Hemptinne et al., 2015; Sanders and Jaeger, 2016). Accordingly, a certain anatomical proximity of the DBS electrode may be necessary for good clinical efficacy of STN-DBS in PD (Akram et al., 2017; Horn et al., 2017; Chen et al., 2018). It remains to be further investigated whether the activity in the upper beta band we recorded from STN and which was coherent to the EMG may mirror motor cortical activity. This hypothesis is corroborated by resting state data showing STN-motor cortical coherence in the upper beta band, whereas STN spectral power was prominent in the lower beta band in patients withdrawn from medication (Litvak et al., 2011).

Our study adds dynamic properties to the complex interplay between STN beta spectral power and its coherence with remote structures of the motor system as a function of movement and dopaminergic state. This may help to identify markers of neuronal activity relevant for more individualized paradigms of DBS therapy.

## Ethics Statement

The study protocol was approved by the ethical review board of the University Hospital Würzburg and patients provided written informed consent to participate prior to implantation.

## Author Contributions

URP: data analysis concept, data analysis and interpretation and creation of figures, writing and revision of the manuscript. FS: conduct of experiments, data interpretation, revision of the manuscript. MR: conduct of experiments, revision of the manuscript. CM: implantation of the DBS systems, revision of the manuscript. JV: study concept, data analysis concept, data interpretation, writing and revision of the manuscript. RR: study concept, data analysis concept, conduct of experiments, data interpretation, writing and revision of the manuscript.

## Conflict of Interest Statement

FS has received speaker and consultant honoraria from Boston Scientific and St. Jude Medical = Abbott and performed research projects sponsored by Boston Scientific and Medtronic. MR has been a member of the advisory board of Medtronic, has received speaking honoraria from Medtronic and grant support from Boston Scientific, St. Jude and TEVA. JV has served as a consultant for Boston Scientific and Medtronic, has received honoraria from Allergan, Merz, UCB, Abbvie, TEVA, Zambon, Bial. RR has received speaking honoraria from Medtronic and travel grants from Medtronic and Boston Scientific. The remaining authors declare that the research was conducted in the absence of any commercial or financial relationships that could be construed as a potential conflict of interest.
